# Predicting Health Disparities in Regions at Risk of Severe Illness to Inform Health Care Resource Allocation During Pandemics: Observational Study

**DOI:** 10.2196/22470

**Published:** 2020-12-02

**Authors:** Tara Fusillo

**Affiliations:** 1 John F Kennedy High School Bellmore, NY United States

**Keywords:** coronavirus, SARS-CoV-2, COVID-19, pandemic, socioeconomic status, predictive model, health care resource allocation

## Abstract

**Background:**

Pandemics including COVID-19 have disproportionately affected socioeconomically vulnerable populations.

**Objective:**

Our objective was to create a repeatable modeling process to identify regional population centers with pandemic vulnerability.

**Methods:**

Using readily available COVID-19 and socioeconomic variable data sets, we used stepwise linear regression techniques to build predictive models during the early days of the COVID-19 pandemic. The models were validated later in the pandemic timeline using actual COVID-19 mortality rates in high population density states. The mean sample size was 43 and ranged from 8 (Connecticut) to 82 (Michigan).

**Results:**

The New York, New Jersey, Connecticut, Massachusetts, Louisiana, Michigan, and Pennsylvania models provided the strongest predictions of top counties in densely populated states with a high likelihood of disproportionate COVID-19 mortality rates. For all of these models, *P* values were less than .05.

**Conclusions:**

The models have been shared with the Department of Health Commissioners of each of these states with strong model predictions as input into a much needed “pandemic playbook” for local health care agencies in allocating medical testing and treatment resources. We have also confirmed the utility of our models with pharmaceutical companies for use in decisions pertaining to vaccine trial and distribution locations.

## Introduction

Socioeconomic vulnerability can directly influence the severity of pandemics and their impact on mortalities in ways like access to health care, household overcrowding, and comorbidities. Prior studies of swine flu (H1N1) have pointed to these factors as contributors to the spread and severity of that pandemic [1]. Other studies have identified national level correlations that are helpful, but not actionable at a local level where actual health care resource allocation decisions are made [2].

Early and accurate decisioning for health care resource allocations are particularly critical in geographic locations with high population density. This research sought to create a repeatable modeling process that uses readily available data sources to identify the top counties in densely populated states with a high likelihood of disproportionate COVID-19 mortality rates.

Stepwise linear regression was used as the modeling technique. Other similar epidemiological research has also used the stepwise linear regression approach including Thomson et al’s [3] 2006 research on environmental models to predict meningitis epidemics in Africa; Chung et al’s [4] 2012 study of the West Nile encephalitis epidemic in Dallas, Texas; Fulton et al’s [5] 2019 predictive models for hospital-based back surgery demand, and Yu et al’s [6] 2005 study on SARS (severe acute respiratory syndrome).

Our objective was to create a repeatable modeling process to identify regional population centers with pandemic vulnerability.

## Methods

Exploratory data research at a national level was performed using county level data (Federal Information Processing Standards [FIPS] for county identification). COVID-19 mortality data sets (deaths per 100,000 people) were created using the Johns Hopkins Dataset [7] and data from the US Census Bureau [8]. Socioeconomic vulnerability data sets at the county level were created using subcomponents of the Centers for Disease Control and Prevention’s (CDC) social vulnerability index (SVI) [9]. The full list of subcomponents can be found in Multimedia Appendix 1.

**Figure 1:**
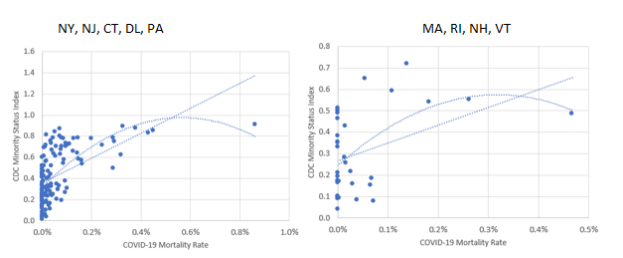
Examples of regional scatterplots and trendlines. CDC: Centers for Disease Control and Prevention.

Scatterplots and trendlines were used to identify variables most correlated with COVID-19 mortalities (see samples in Figure 1). Few, if any, social vulnerability variables correlated across all of the 3142 FIPS counties, but minority status correlated strongly in certain regions, particularly those with high mortality rates. These initial findings led the author to focus the next phase of research and modeling on state level rather than national level correlations. County-level dependent variable data sets were created using COVID-19 mortality and population data from the Corona Data Scraper website (data service that scrapes county level COVID-19 data on a daily basis) [10] as well as from USAFacts [11] for cumulative mortalities as of April 8, 2020, and May 8, 2020, respectively.

County-level independent variable data sets were created using socioeconomic data from the County Health Rankings website, a collaboration data set created by the Robert Wood Johnson Foundation and the University of Wisconsin Population Health Institute [12]. Given the year to year stability of most of these socioeconomic variables, the latest data available from the County Health Rankings website was used, and no attempt was made to augment the data set to try to match the time-series to either April 8, 2020, or May 8, 2020. The full list of independent variables available in this data set can be found in Multimedia Appendix 1.

Cumulative COVID-19–specific mortality data (deaths per 100,000 people) by county for states with a high mortality rate (New York, New Jersey, Connecticut, Massachusetts, Louisiana, Michigan, and Pennsylvania) as of May 8 was used as the dependent variable [13]. The full ranking of states by mortality rate can be found in Multimedia Appendix 1.

**Figure 2:**
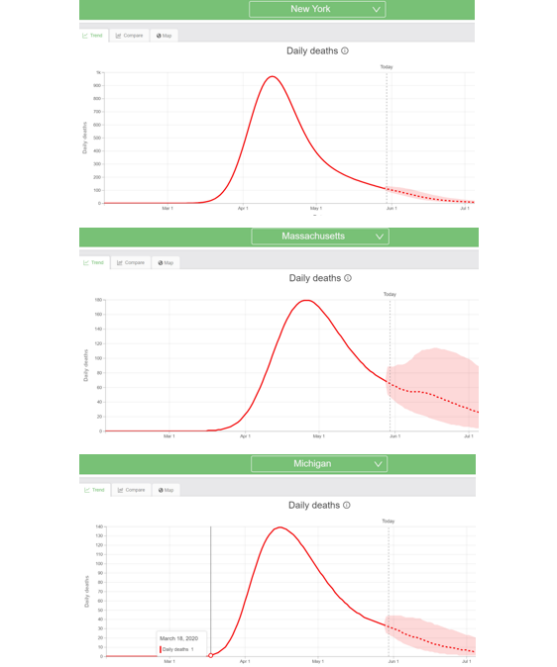
Examples of COVID-19 mortality curves from the Institute for Health Metrics and Evaluation [14].

The May 8 date was used to ensure that the dependent variable would be tuned to a timeframe at or around the peak in daily mortalities when health care resources (testing, treatment, and tracing) were typically most needed. The mortality curves in Figure 2 provide support for May 8 as the overall date for mortality predictions as shown in the Institute for Health Metrics and Evaluation data set [14].

A stepwise linear regression technique was used to build each state level model. All relevant independent variables were initially used in the model (ie, include severe housing problems, but exclude violent deaths). Next, the variable with the lowest T-statistic was removed and the linear regression was rerun. This process was repeated until all T-statistics for the remaining independent variables were near a value of 2 or greater.

## Results

Predictive models were completed for New York, New Jersey, Connecticut, Massachusetts, Louisiana, Michigan, and Pennsylvania, with statistically significant results. The final list of model variables, coefficients, variable correlations, sample sizes, and *P* values can be found in Multimedia Appendix 1.

**Figure 3:**
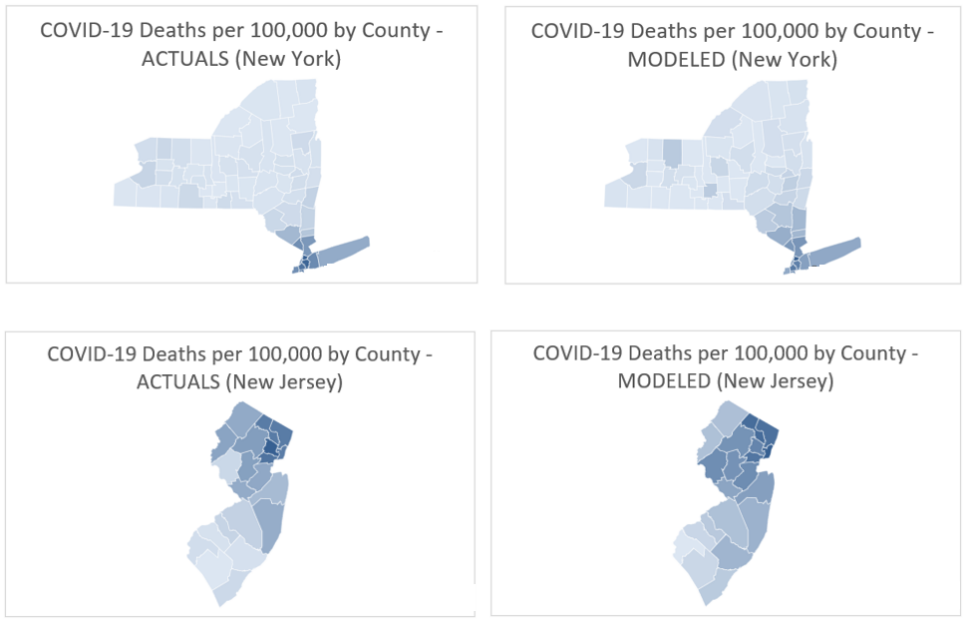
COVID-19 mortality rate heatmaps for New York and New Jersey (actuals vs model prediction) using Microsoft Excel mapping tools.

Model validation comparing predicted to actual county rankings by cumulative mortality rate (deaths per 100,000 people) through May 8 (see Tables 1 and 2 for New York and New Jersey), and data visualizations using state-level maps were completed (Figure 3). These validations were used to share model methods and results with each state’s Department of Health Commissioner and, where appropriate, with an outside agency.

**Table 1:** Top counties by mortality rate for New York (actuals vs model prediction). Both actual values and modeled predictions indicate that these counties had high mortality rates relative to other counties within the state.

New York county	Deaths per 100,000 people (5/8 actuals), n	Deaths per 100,000 people (5/8 model), n
Bronx	198	197
Kings	167	155
Queens	188	148
Westchester	115	118
New York	104	118
Rockland	129	114
Nassau	134	102
Suffolk	87	91
Richmond	128	88
Orange	68	83

**Table 2:** Top counties by mortality rate for New Jersey (actuals vs model prediction). Both actual values and modeled predictions indicate that most of these counties had high mortality rates relative to other counties within the state (exception marked with “a”).

New Jersey county	Deaths per 100,000 people (5/8 actuals), n	Deaths per 100,000 people (5/8 model), n
Hudson	140	139
Passaic	143	126
Bergen	143	121
Union	152	116
Essex	175	101
Middlesex	91	93
Hunterdon	35^a^	93
Somerset	100	88
Morris	103	88

^a^Indicates low mortality rate relative to all other counties within the state.

Four further validations were completed. The first validation was to check model performance using COVID-19 mortality data on April 8, 2020, instead of May 8, 2020. This validation tested whether models using data available early in the pandemic would have been sufficient to make accurate predictions. The same variables were used, but coefficients were recalibrated with the April 8 data set. The April 8 and May 8 model outputs were compared to test for stability in the top counties predicted for high COVID-19 mortality rates. For New York, New Jersey, and Connecticut, the models proved to be stable. For Massachusetts, the 4/8 model performance was not stable, but this was easily corrected by using case data in the place of mortality data. This is an important finding as it validates the predictive power contained in early case data, which is more readily available at the start of a pandemic. The New York and Massachusetts model validation result summaries can be found in Multimedia Appendix 1.

The second validation was to check model performance beyond May 8, 2020 (ie, using the May 8 model to predict July 31 mortalities). Results were less stable as most states began their reopenings in mid-May creating differential effects by county. However, the models for New York, New Jersey, Connecticut, and Massachusetts continued to identify the counties with the highest cumulative mortality rates.

The third validation was to check the independent variables for multicollinearity, with a specific focus on the correlations between “Black” and variables such as “severe housing” and “uninsured.” Strong multicollinearity was seen in Connecticut, Massachusetts, Louisiana, and Michigan partially explaining why these variables did not remain in the model after the stepwise regression process. Multicollinearity results for these states are presented in Multimedia Appendix 1. Future models could consider composite variables to address this multicollinearity and to maintain combined effects such as “Black,” “severe housing,” and “uninsured.”

The fourth validation leveraged an out-of-sample methodology. For New York, New Jersey, Connecticut, and Massachusetts, only one half of the data points (ie, half of the counties in each state) were used to build the model. Coefficients were recalibrated and variables were removed if *T* values fell below 2. In each state, the out-of-sample model continued to identify the top counties for cumulative mortality rates through May 8.

With models and validations completed, the Departments of Health for New York, New Jersey, Connecticut, Massachusetts, Louisiana, Michigan, and Pennsylvania were contacted (Table 3). Additionally, agents of the New York Department of Health (Northwell Health and CORE), a third-party statistical modeling firm for Connecticut (COVIDACTNOW), and a third-party modeling firm for Pennsylvania (Mathematica) were contacted. Response from these contacts were positive and, in some cases, occurred within an hour of outreach (Northwell Health). This response indicates the strong need for this type of health care resource allocation tool for pandemics and other health crises. In fact, the Pennsylvania Department of Health indicated this tool’s importance in a second wave of COVID-19.

The final data sets for our New York, New Jersey, Connecticut, Massachusetts, Louisiana, Michigan, and Pennsylvania models are contained in Multimedia Appendix 1.

**Table 3:** State Department of Health (DOH) contact summary.

DOH or outside agency	Models shared	Receipt accepted	Zoom session
New York DOH	✓	✓	
Northwell Health (New York)	✓	✓	
CORE (New York)	✓		
New Jersey DOH	✓	✓	
Connecticut DOH	✓	✓	
COVIDACTNOW (Connecticut)	✓	✓	
Massachusetts DOH	✓		
Michigan DOH	✓		
Louisiana DOH	✓		
Pennsylvania DOH	✓	✓	✓
Mathematica	✓	✓	✓

## Discussion

### Principal Findings

The research described in this paper has shown the extent to which Black Americans, people living in crowded housing units, and households with less access to health care are at higher risk of severe illness during a pandemic. The results of other studies provide several possible explanations for these findings. Individuals with lower income are more likely to live in crowded housing units and multifamily homes [15]. Lower income households are also structurally disadvantaged in their access to medical insurance and health care [16]. Some studies have also pointed to a concept called “weathering” within the Black American community. Arline Geronimus, a professor of public health at the University of Michigan, showed through her research that among Black communities, coping with financial strain, discrimination, and barriers to good education elevates the stress response, contributing to obesity, diabetes, hypertension, and heart disease [17].

The CDC conducted research on the co-occurrence of COVID-19 and certain ethnicities using a sample of 580 patients with lab-confirmed data [18]. Their results showed more hospitalizations in Black patients than White patients. The researchers cited underlying medical conditions, work circumstances, and living conditions to be major factors in COVID-19 mortalities in their sample. For example, members of racial and ethnic minorities were more likely to live in densely populated areas, making it more difficult to practice social distancing and being more susceptible to contracting and spreading COVID-19. These members also lived farther away from grocery stores and medical facilities, thus being less able to receive necessary resources and medical attention. Other examples cited included Hispanic and Black American workers employed in higher-risk industries and often lacking paid sick leave. The researchers also hypothesized that these types of workers were more likely to continue working despite being sick, thus exposing other workers to the disease. The CDC recommended at the conclusion of this research that public health officials communicate to different population groups about COVID-19 and provide more health care services to ethnic minority groups.

While such studies are insightful, we are not aware of any research that translates these impacts into predictive models that can be used to direct local health care resources to the communities most likely to need them, thereby reducing mortalities caused by an ongoing set of institutional inequities.

That said, some organizations have attempted to create health care resource allocation methods using descriptive statistics. The CDC created the SVI, allowing health care communities to see which factors contribute to socioeconomic vulnerability. The CDC SVI factors are grouped into four groups: Socioeconomic Status, Household Composition & Disability, Minority Status & Language, and Housing Type & Transportation. Although these factors are crucial inputs in identifying specific vulnerable communities, these four categories of factors alone are not sufficient to create the types of predictive models that state and local health care agencies can use. One example of this insufficiency is a recent study at Emory University where researchers identified correlations between COVID-19 mortalities and the SVI at a national level in the United States [2]. While this study is valuable, it stopped short of recommending methods or processes to effectively distribute health care resources to specific counties in the United States, particularly in the early days of the pandemic. Another study from the Surgo Foundation stated that COVID-19 created new challenges for many communities tied to health and structural factors that were not completely captured by the CDC SVI [19]. In addition to the four socioeconomic factors provided by the CDC, the Surgo Foundation added two more factors: Epidemiologic Factors and Healthcare System Factors. They stated that underlying health conditions in addition to health care system factors have been proven to greatly increase a community’s vulnerability during a pandemic. The Surgo Foundation combined these two factors with the CDC SVI to create the COVID-19 Community Vulnerability Index (CCVI). The Surgo Foundation created heatmaps to show retrospectively which counties were most vulnerable as measured by their CCVI. Similar to the Emory University study, however, this methodology did not create a predictive model to identify where the mortalities would be highest at peak periods in a pandemic. We also compared the Surgo Foundation heatmap to our own predictive model rankings and confirmed that our projections of the top counties by per capita mortalities were far closer to actual peaks. Results of this comparison are shown in Multimedia Appendix 1.

During the early phases of the research described in this paper, we used the CDC’s SVI, similar to the Emory University study. We explored all of the subcategory factors in the SVI, but none showed strong correlations at a FIPS county level across the United States. We then grouped states together by region and found strong correlations in the most densely populated regions, particularly with the minority status subfactors. Similar to the Surgo Foundation study, we posited that the CDC’s SVI subfactors alone would be insufficient to build predictive models, so a far more complete independent variable data set of socioeconomic and health care data was sourced from the County Health Rankings website as discussed earlier. The key differentiation of our work is the predictive models for each state given the local differences in how each of the factors act as predictors of peak COVID-19 per capita mortalities. Our experience in meeting with the Pennsylvania Department of Health in early June 2020 confirms the uniqueness and usefulness of the approach given that they will be using the predictive modeling process for health care resource allocations in the event of a potential second wave of COVID-19 in Fall 2020. Other state-level departments of health and agents of these governmental functions were similarly intrigued by our predictive approach including those in New York, New Jersey, and Connecticut. Finally, the Chief Information Officer of Johnson & Johnson has forwarded the author’s research to J&J’s Health and Human Services Group for possible use in decisions pertaining to vaccine trial and distribution locations.

### Limitations

A number of limitations must be acknowledged. The models employed in the analyses are reliant on the accuracy of the data sets compiled. COVID-19 mortality data in particular has been notoriously difficult for states to report accurately at a county level throughout the pandemic for reasons including mortality cause classification errors at the offices of the local coroner [20]. This systemic undercounting could have created some correlations between our variables and reporting errors. That said, if reporting errors are similar across counties within a state, then these unwanted effects to our model are likely to be small since we created a different model for each state.

The models are only valid within the range of county-level data for the following states: New York, New Jersey, Connecticut, Massachusetts, Louisiana, Michigan, and Pennsylvania. Models would need to be rebuilt and validated for each additional state. In addition, models for states with lower levels of per capita mortalities were far less predictive, although health care resource allocations would be less critical in those regions.

In addition, we would note that while we did not find significant correlations between the subcomponents of the CDC SVI and COVID-19 mortalities, other data sets at the FIPS level (eg, American Community Survey data and census data) might yield different results. For example, there may be variables that are correlated with one another that also correlate with COVID-19 mortalities. Gore et al [21] showed the difficulties in teasing apart specific population demographic measures at a granular level into a linear regression model since so many of these variables correlate highly with one another.

### Conclusions

Our modeling process can be used for the early identification of the communities most in need of health care resources during future pandemics or health crises. The COVID-19 models and the overall methodology have been received with enthusiasm by the New York, New Jersey, Connecticut, Massachusetts, Louisiana, Michigan, and Pennsylvania Departments of Health. All Commissioners responded positively, which validates our hypothesis that neither other researchers nor the Departments of Health themselves have developed a similar modeling process as a means to allocate scarce health care resources such as testing, treatment, tracing, education, and communication. We have also confirmed the utility of our models with pharmaceutical companies for use in vaccine trial and distribution location decisions.

Future modeling processes could also include building hierarchical models to improve county rankings by better accounting for effects of clustered variables similar to Fulton et al [5] in their 2019 models for predicting hospital-based back surgery by geography. The gradient boosting approach leveraged by Fulton et al [5] may also be useful to examine states with lower population densities where our stepwise linear regression models proved to be weaker. Finally, the group-personalized regression approach pioneered by Palmius et al [22] in their 2018 models for predicting mental health scores by group rather than for an overall population could also be explored.

Other research papers evaluated during the course of this research and other data sets referenced in this research can be found in Multimedia Appendix 2.

## Appendix

Multimedia Appendix 1 Supplemental materials.

Multimedia Appendix 2 Other data sets referenced in this research and research papers evaluated during the course of this research.

## Abbreviations

CCVI: COVID-19 Community Vulnerability Index
CDC: Centers for Disease Control and Prevention
FIPS: Federal Information Processing Standards
SARS: severe acute respiratory syndrome
SVI: social vulnerability index
