# Supplementary material for: Predicting Health Disparities in Regions at Risk of Severe Illness to Inform Health Care Resource Allocation During Pandemics: Observational Study
Source: JMIRx Med. 2020 Dec 2;1(1):e22470. doi: 10.2196/22470 (PMC7924701; doi:10.2196/22470)
Supplement: Multimedia Appendix 1 [file med_v1i1e22470_app1.pdf]

This is a Multimedia Appendix to a full manuscript published in the JMIR COVID-19 Journal

Predicting Health Disparities in Regions at Risk of Severe Illness to inform Healthcare Resource Allocations during Pandemics: Observational Study

Tara Fusillo
John F. Kennedy High School
3000 Bellmore Avenue
Bellmore, NY 11710
Phone: 516-521-3874
Fax: 516-992-2396
tfushiro@gmail.com

Figure 6: Full list of sub-component variables from the CDC

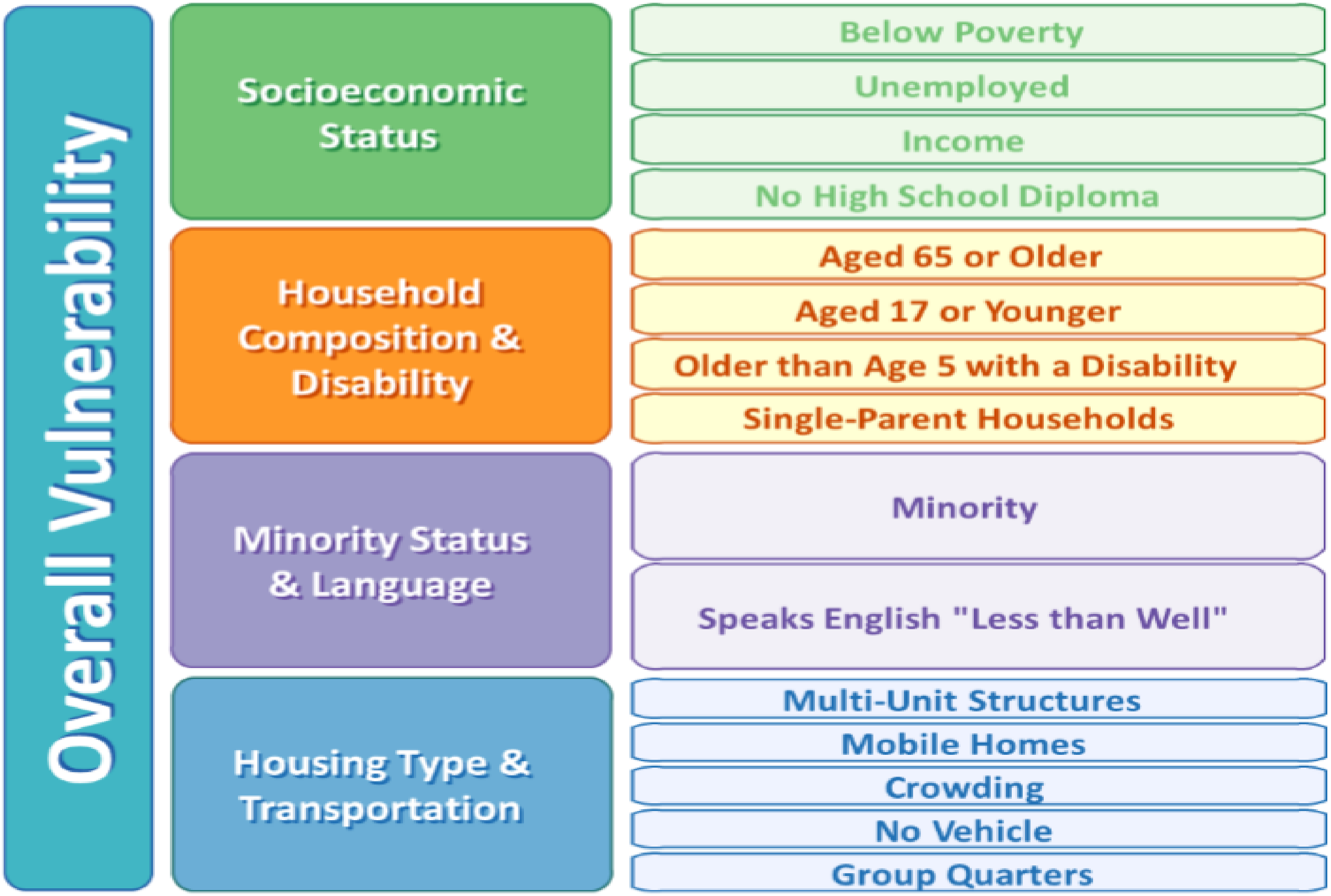

Figure 7: Full variable list from countyhealthrankings.org

2020 Ranked Measures & Data Sources

	Measure	*Source*	*Years of Data*
**HEALTH OUTCOMES**			
Length of Life	Premature death (50%)	National Center for Health Statistics – Mortality files	2016-2018
Quality of Life	Poor or fair health (10%)	Behavioral Risk Factor Surveillance System	2017
	Poor physical health days (10%)	Behavioral Risk Factor Surveillance System	2017
	Poor mental health days (10%)	Behavioral Risk Factor Surveillance System	2017
	Low birthweight (20%)	National Center for Health Statistics – Natality files	2012-2018
**HEALTH FACTORS**			
**HEALTH BEHAVIORS**			
Tobacco Use	Adult smoking (10%)	Behavioral Risk Factor Surveillance System	2017
Diet and Exercise	Adult obesity (5%)	United States Diabetes Surveillance System	2016
	Food environment index (2%)	USDA Food Environment Atlas, Map the Meal Gap	2015 & 2017
	Physical inactivity (2%)	United States Diabetes Surveillance System	2016
	Access to exercise opportunities (1%)	Business Analyst, Delorme map data, ESRI, & US Census Tigerline Files	2010 & 2019
Alcohol and Drug Use	Excessive drinking (2.5%)	Behavioral Risk Factor Surveillance System	2017
	Alcohol-impaired driving deaths (2.5%)	Fatality Analysis Reporting System	2014-2018
Sexual Activity	Sexually transmitted infections (2.5%)	National Center for HIV/AIDS, Viral Hepatitis, STD, and TB Prevention	2017
	Teen births (2.5%)	National Center for Health Statistics – Natality files	2012-2018
**CLINICAL CARE**			
Access to Care	Uninsured (5%)	Small Area Health Insurance Estimates	2017
	Primary care physicians (3%)	Area Health Resource File/American Medical Association	2017
	Dentists (1%)	Area Health Resource File/National Provider Identification file	2018
	Mental health providers (1%)	CMS, National Provider Identification file	2019
Quality of Care	Preventable hospital stays (5%)	Mapping Medicare Disparities Tool	2017
	Mammography screening (2.5%)	Mapping Medicare Disparities Tool	2017
	Flu vaccinations (2.5%)	Mapping Medicare Disparities Tool	2017
**SOCIAL AND ECONOMIC FACTORS**			
Education	High school graduation (5%)	EDFacts & State-specific sources	Varies
	Some college (5%)	American Community Survey	2014-2018
Employment	Unemployment (10%)	Bureau of Labor Statistics	2018
Income	Children in poverty (7.5%)	Small Area Income and Poverty Estimates	2018
	Income inequality (2.5%)	American Community Survey	2014-2018
Family and Social Support	Children in single-parent households (2.5%)	American Community Survey	2014-2018
	Social associations (2.5%)	County Business Patterns	2017
Community Safety	Violent crime (2.5%)	Uniform Crime Reporting – FBI	2014 & 2016
	Injury deaths (2.5%)	National Center for Health Statistics – Mortality files	2014-2018
**PHYSICAL ENVIRONMENT**			
Air and Water Quality	Air pollution – particulate matter[1] (2.5%)	Environmental Public Health Tracking Network	2014
	Drinking water violations (2.5%)	Safe Drinking Water Information System	2018
Housing and Transit	Severe housing problems (2%)	Comprehensive Housing Affordability Strategy (CHAS) data	2012-2016
	Driving alone to work (2%)	American Community Survey	2014-2018
	Long commute – driving alone (1%)	American Community Survey	2014-2018

Figure 8: COVID-19 mortality rates by state as May 8, 2020 from statista.com

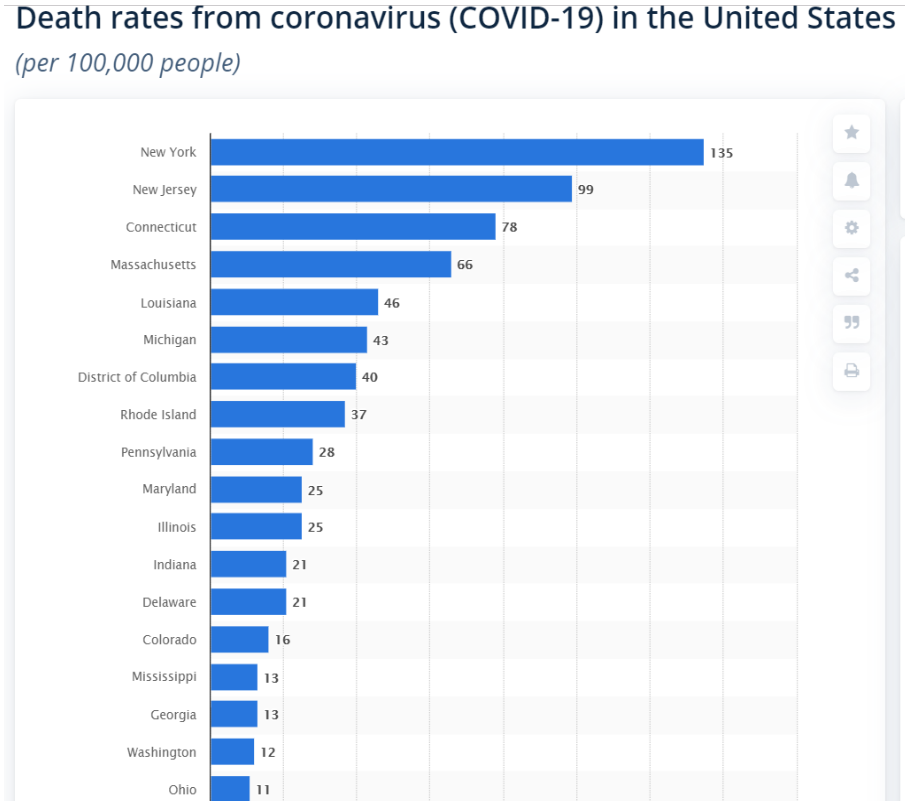

Figure 9: Final model variables

	Top States by COVID-19 Mortality								Other States				
	NY	NJ	CT	MA	LA	MI	PA		CA	CA	FL	TX	TX
Dependent Variable													
Deaths per 100,000 Population	X	X	X	X	X	X	X		X		X	X	
Cases per 100,000 Population										X			X
Independent Variable (T-Statistic)													
Demographics													
**% Black**	2.1	1.3	3.1	2.5	5.0	7.7						0.8	
**% Hispanic**	2.1				2.8				2.8	1.7			
**% Asian**										3.4		1.5	3.6
**% 65 and over**											2.6		
% White													
Socioeconomics													
**Median Household Income**	2.2					1.6	3.8		2.9				
**% Severe Housing Problems** *(multi-family)*	4.0	2.9					4.8				2.5		
% Uninsured													
**Average Daily PM2.5** *(Pollution)*				4.8						1.8			
% Unemployed													
Health													
% Adults with Obesity													
**Primary Care Access (Z-Score)**					1.9								
**Life Expectancy** *(comorbidities)*	1.6	5.1	1.9		1.9								
% Limited Access to Healthy Foods													

Figure 10: Correlations, Sample Size & P-Values

	Correlations to COVID-19 Mortalities						
	NY	NJ	CT	MA	LA	MI	PA
Demographics							
% Black	80%	24%	78%	36%	38%	65%	43%
% Hispanic	87%	56%	61%	44%	20%	7%	49%
% Asian	75%	40%	61%	52%	21%	39%	56%
% 65 and over	-46%	-46%	-2%	-45%	-3%	-32%	-29%
Socioeconomics							
Median Household Income	49%	19%	39%	2%	20%	19%	48%
% Severe Housing Problems *(multi-family)*	90%	52%	64%	-17%	21%	23%	57%
% Uninsured	55%	45%	54%	-41%	-26%	-28%	-12%
Average Daily PM2.5 *(Pollution)*		-21%	64%	75%	-17%	33%	-15%
% Unemployed	-29%	-34%	-3%	-49%	-23%	-19%	-3%
Health							
% Adults with Obesity	-61%	-49%	-69%	-69%	-3%	-6%	-39%
Primary Care Access (Z-Score)	-40%	-10%	-93%	54%	4%	-18%	-31%
Life Expectancy *(comorbidities)*	70%	62%	60%	-22%	12%	-17%	11%
% Limited Access to Healthy Foods	-52%	-73%	-60%	-10%	0%	-2%	-17%
Number of Data Points	62	21	8	14	64	83	49
P value	0.00000	0.00008	0.02380	0.00093	0.00008	0.00000	0.00000

Figure 11: New York model validation results example using April 8 mortality data

New York Counties	Deaths per 100,000 (5/8)	Deaths per 100,000 (5/8 MODEL)
Bronx	198	197
Kings County, NY	167	155
Queens County, NY	188	148
Westchester	115	118
New York	104	118
Rockland	129	114
Nassau	134	102
Suffolk	87	91
Richmond County, NY	128	88
Orange	68	83

New York Counties	Deaths per 100,000 (5/8)	Deaths per 100,000 (4/8 MODEL)
Bronx	198	68
Queens County, NY	188	47
Kings County, NY	167	46
New York	104	38
Westchester	115	37
Rockland	129	34
Nassau	134	28
Richmond County, NY	128	26
Suffolk	87	26
Orange	68	25

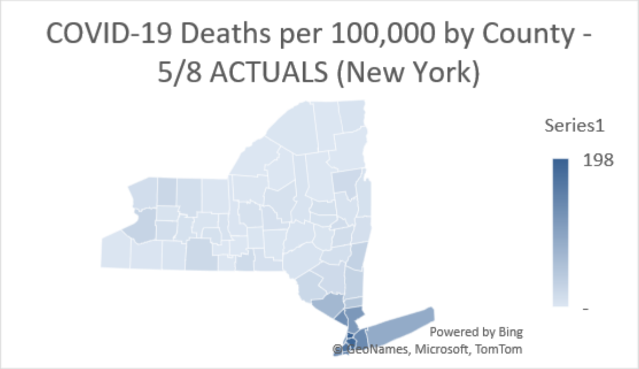

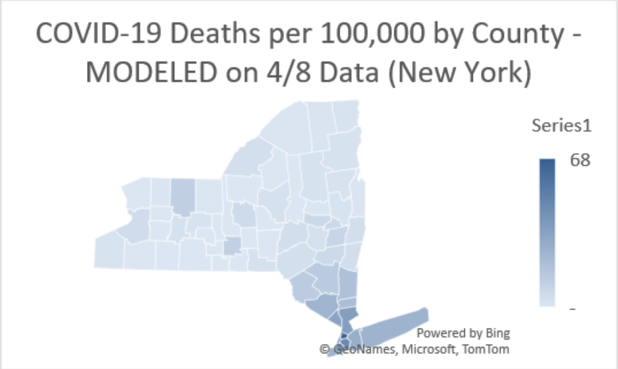

Figure 12: Massachusetts model validation results example using April 8 mortality data

Massachusetts County	Deaths per 100,000 (ACTUALS)	Deaths per 100,000 (MODEL)
Suffolk County	75.8	83.3
Middlesex County	63.9	73.4
Hampden County	88.6	70.9
Norfolk County	81.8	66.6
Worcester County	39.9	55.8
Hampshire County	29.2	54.4

Massachusetts County	Deaths per 100,000 (ACTUALS - 5/8)	Deaths per 100,000 (MODEL - 4/8)
Middlesex County	63.9	10.4
Hampshire County	29.2	10.1
Franklin County	52.4	10.0
Essex County	66.9	9.6
Berkshire County	28.6	9.4
Worcester County	39.9	9.4

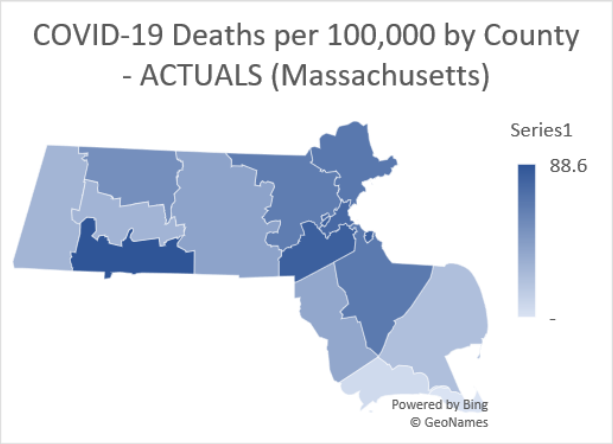

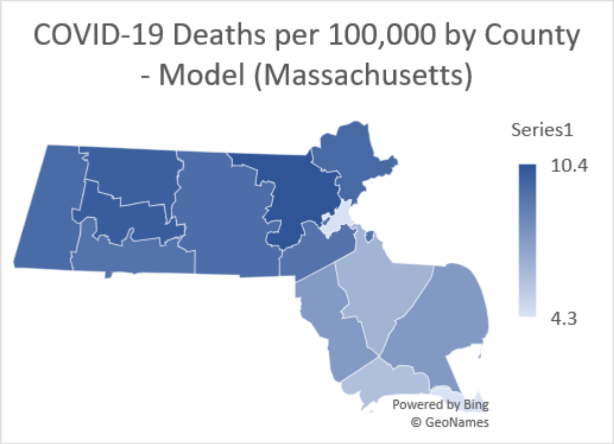

Figure 13: Massachusetts model validation results example using April 8 case data

Massachusetts County	Deaths per 100,000 (ACTUALS)	Deaths per 100,000 (MODEL)
Suffolk County	75.8	83.3
Middlesex County	63.9	73.4
Hampden County	88.6	70.9
Norfolk County	81.8	66.6
Worcester County	39.9	55.8
Hampshire County	29.2	54.4

Massachusetts County	Deaths per 100,000 (ACTUALS - 5/8)	Cases per 100,000 (MODEL - 4/8)
Suffolk County	75.8	406.9
Hampden County	88.6	267.7
Norfolk County	81.8	252.1
Middlesex County	63.9	251.7
Plymouth County	65.8	245.2
Worcester County	39.9	206.5

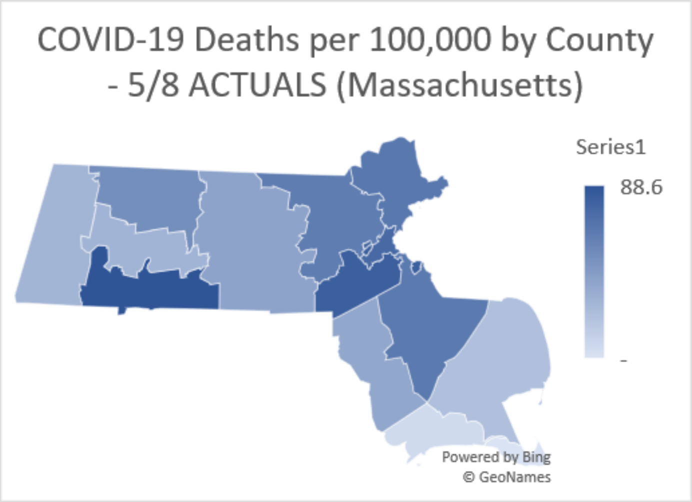

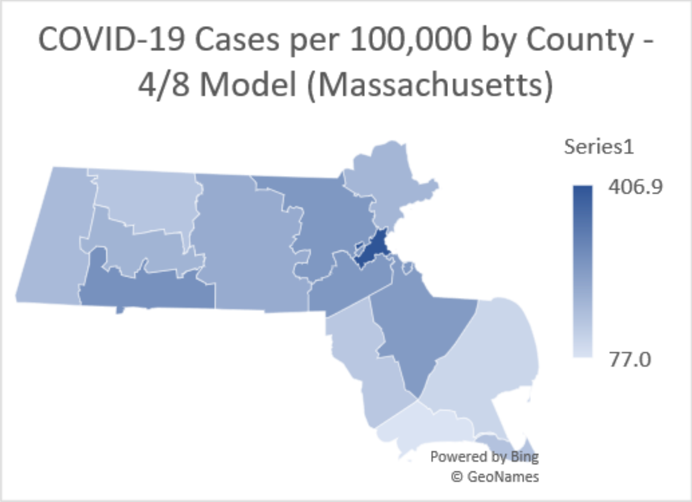

Figure 14: Multi-collinearity results for states without severe housing and other socioeconomically vulnerable variables

		Top States by COVID-19 Mortality			
		CT	MA	LA	MI
Correlation		Black	Black	Black	Black
Severe Housing		75%	56%	58%	45%
Uninsured		51%			
Primary Care			33%		
Unemployed				44%	
Obesity					

Figure 15: Comparison to Surgo Foundation Heatmap CCVI rankings

Pennsylvania Counties	Deaths per 100,000 (ACTUAL) - May 8th	Deaths per 100,000 (MODEL)	CCVI (Surgo)
Philadelphia	4.0	4.4	0.80
Chester	2.9	4.3	0.15
Monroe	3.5	4.0	0.68
Bucks	4.5	4.0	0.14
Montgomery	5.3	3.7	0.18
Delaware	5.6	3.5	0.36
Lehigh	2.6	3.2	0.27

New York Counties	Deaths per 100,000 (5/8)	Deaths per 100,000 (5/8 MODEL)	CCVI (Surgo)
Bronx	198	197	0.99
Kings County, NY	167	155	0.91
Queens County, NY	188	148	0.83
Westchester	115	118	0.52
New York	104	118	0.32
Rockland	129	114	0.56
Nassau	134	102	0.17
Suffolk	87	91	0.28
Richmond County, NY	128	88	0.58
Orange	68	83	0.60

New Jersey Counties	Deaths per 100,000 (ACTUALS 5/8)	Deaths per 100,000 (MODEL 5/8)	CCVI (Surgo)
Hudson County	139.8	138.6	0.64
Passaic County	142.5	125.9	0.40
Bergen County	142.6	121.4	0.08
Union County	151.7	116.2	0.45
Essex County	175.0	100.9	0.52
Middlesex County	90.7	93.4	0.38
Hunterdon County	35.4	92.8	0.06
Somerset County	99.7	88.4	0.08
Morris County	102.9	87.7	0.10

Massachusetts Counties	Deaths per 100,000 (ACTUALS)	Deaths per 100,000 (MODEL)	CCVI (Surgo)
Suffolk County	75.8	83.3	0.31
Middlesex County	63.9	73.4	0.30
Hampden County	88.6	70.9	0.38
Norfolk County	81.8	66.6	0.16
Worcester County	39.9	55.8	0.33
Hampshire County	29.2	54.4	0.10

Figure 16: New York Datasets

New York Counties	Deaths per 100,000 (ACTUALS)	% Black	% Hispanic	Median Household Income	% Severe Housing Problems	Life Expectancy	Deaths per 100,000 (MODEL)	Absolute Difference (Model - Actuals)
	100	1.3	1.1	0.0	4.0	3.6	94.7	11.0
Albany	14	12.8	6.1	64536	16	80.0	34	20.6
Allegany	4	1.3	1.7	46262	14	78.0	-	4.3
Bronx	198	29.2	56.4	38566	39	80.6	197	1.4
Broome	9	5.5	4.3	50928	16	78.7	11	2.4
Cattaraugus	3	1.4	2.1	48017	15	77.8	-	2.6
Cayuga	3	4.0	3.0	52945	12	79.4	-	2.6
Chautauqua	2	2.1	7.8	45689	16	77.3	5	3.3
Chemung	1	6.0	3.2	52565	14	77.2	-	1.2
Chenango	2	0.9	2.2	50312	14	77.3	-	2.1
Clinton	5	4.0	3.0	55517	14	79.4	8	2.9
Columbia	30	4.7	4.9	64916	15	79.6	18	12.0
Cortland	2	1.7	2.7	51751	14	78.2	-	2.1
Delaware	4	1.7	4.0	47382	17	79.3	12	7.4
Dutchess	29	10.5	12.5	79604	21	80.6	68	39.0
Erie, NY	27	13.1	5.7	56375	15	78.4	22	5.3
Essex, NY	-	2.3	3.0	54376	13	80.8	6	6.5
Franklin, NY	-	5.6	3.6	48722	13	79.3	3	3.1
Fulton	9	2.0	3.2	51388	14	78.4	-	9.3
Genesee	5	2.8	3.4	58925	12	78.1	-	5.2
Greene	11	5.6	6.0	54514	18	78.6	26	15.3
Hamilton, NY	-	0.9	1.7	59020	16	79.9	11	11.1
Herkimer, NY	5	1.2	2.2	53168	11	79.2	-	4.8
Jefferson, NY	-	6.2	7.8	52798	14	78.6	11	11.0
Kings County, NY	167	29.9	19.1	60862	34	82.6	155	12.1
Lewis County NY	-	0.7	1.8	52660	12	79.5	-	-
Livingston County, NY	8	2.5	3.7	61027	14	80.0	12	3.8
Madison	8	1.7	2.3	59114	12	80.9	0	8.0
Monroe	12	14.6	9.0	60240	16	79.9	41	28.9
Montgomery	8	2.0	14.9	43799	17	77.7	17	8.6
Nassau	134	11.6	17.2	115301	21	82.9	102	31.7
New York	104	12.5	25.9	84610	24	84.9	118	14.6
Niagara County, NY	13	7.0	3.1	55013	13	77.7	-	12.8
Oneida	7	6.1	6.1	54096	15	78.4	12	4.3

New York Counties	Deaths per 100,000 (ACTUALS)	% Black	% Hispanic	Median Household Income	% Severe Housing Problems	Life Expectancy	Deaths per 100,000 (MODEL)	Absolute Difference (Model - Actuals)
Onondaga	8	10.9	5.1	59786	15	79.5	24	16.0
Ontario	4	2.3	5.0	62930	13	79.8	7	3.8
Orange	68	10.4	21.0	75048	23	80.2	83	14.6
Orleans	22	5.9	4.9	52658	15	77.7	6	15.9
Oswego	2	1.0	2.7	53745	16	78.1	3	1.5
Otsego	5	2.0	3.8	54321	16	79.8	15	9.9
Putnam	46	2.8	15.8	100887	18	82.5	69	22.9
Queens County, NY	188	18.0	28.1	68432	32	83.8	148	40.4
Rensselaer	10	6.9	5.2	71084	14	79.0	20	9.9
Richmond County, NY	128	9.4	18.7	79719	24	80.9	88	40.0
Rockland	129	11.4	18.1	88960	27	83.2	114	14.6
St. Lawrence	2	2.3	2.4	49356	15	79.1	4	2.1
Saratoga	6	1.7	3.4	83210	11	81.0	12	5.9
Schenectady	15	10.8	7.4	63297	15	79.0	27	12.5
Schoharie	3	1.3	3.3	57757	15	80.7	12	8.5
Schuyler	-	1.0	1.9	50190	15	78.1	-	-
Seneca	6	4.9	3.5	54184	13	77.5	-	5.8
Steuben	20	1.5	1.7	54862	12	78.7	-	19.6
Suffolk	87	7.4	19.8	100075	23	80.7	91	3.6
Sullivan	21	8.2	16.6	51985	19	77.9	39	18.1
Tioga	16	0.9	2.1	60309	11	80.4	-	16.3
Tompkins	-	4.0	5.3	57383	20	80.7	40	39.6
Ulster	17	6.1	10.5	63073	20	79.8	47	29.9
Warren	17	1.2	2.7	56694	16	79.3	10	7.2
Washington	8	2.9	2.8	55913	15	78.6	5	2.6
Wayne	1	2.8	4.5	61118	12	78.7	-	1.1
Westchester	115	13.8	25.1	94521	24	83.5	118	3.3
Wyoming	12	5.1	3.3	57709	9	78.7	-	12.3
Yates	8	0.9	2.3	51089	16	78.2	2	6.2

Regression Statistics								
Multiple R	0.943128							
R Square	0.88949							
Adjusted R Square	0.879624							
Standard Error	17.02748							
Observations	62							
ANOVA								
	df	*SS*	*MS*	*F*	*Significance F*			
Regression	5	130686.5	26137.29	90.14872	1.64894E-25			
Residual	56	16236.37	289.9352					
Total	61	146922.8						
% Black	1.343715	0.625107	2.149576	0.035924	0.091475472	2.595955	0.091475	2.595955
% Hispanic	1.147654	0.536479	2.139235	0.03679	0.072957804	2.222349	0.072958	2.222349
Median Household Income	0.000466	0.000216	2.159238	0.03513	3.36697E-05	0.000898	3.37E-05	0.000898
Life Expectancy	3.602722	2.216237	1.625603	0.109649	-0.836934501	8.042378	-0.83693	8.042378

Figure 17: New Jersey Datasets

County	Deaths per 100,000 (ACTUALS)	Life Expectancy	% Severe Housing Problems	% Black	Deaths per 100,000 (MODEL)	Absolute Difference (Model - Actuals)
	81.9	**14.2**	**4.4**	-		21.7
**(1,156.7)**						
Atlantic County	25.8	77.2	25	14.6	52.9	27.1
Bergen County	142.6	83.2	21	5.5	121.4	21.2
Burlington County	42.7	79.4	16	16.7	41.2	1.4
Camden County	39.3	77.3	20	18.6	32.9	6.4
Cape May County	33.7	77.2	20	4.1	31.6	2.1
Cumberland County	18.7	75.4	23	18.6	19.0	0.2
Essex County	175.0	79.5	29	38.6	100.9	74.0
Gloucester County	24.7	77.8	16	10.4	19.7	4.9
Hudson County	139.8	82.2	29	10.7	138.6	1.2
Hunterdon County	35.4	83.4	14	2.5	92.8	57.4
Mercer County	87.6	80.5	19	19.6	69.6	18.0
Middlesex County	90.7	81.5	21	9.7	93.4	2.7
Monmouth County	69.3	80.5	20	6.8	75.2	5.8
Morris County	102.9	82.6	16	3.2	87.7	15.1
Ocean County	85.0	79.1	20	3.0	56.3	28.7
Passaic County	142.5	80.7	31	10.2	125.9	16.6
Salem County	28.9	76.2	18	13.4	4.0	24.8
Somerset County	99.7	82.5	16	9.4	88.4	11.3
Sussex County	89.0	79.4	16	2.1	42.7	46.3
Union County	151.7	81.5	26	20.9	116.2	35.5
Warren County	95.9	79.4	16	4.8	41.7	54.3

Regression Statistics	
Multiple R	0.84221
R Square	0.70932
Adjusted R Square	0.65803
Standard Error	28.2249
Observations	21

ANOVA

	df	*SS*	*MS*	*F*	*gnificance F*
Regression	3	33048	11016	13.828	8.1E-05
Residual	17	13543	796.647		
Total	20	46591			

	Coefficients	*andard Err*	*t Stat*	*P-value*	*Lower 95%*	*Jpper 95%*	*ower 95.0%*	*pper 95.0%*
Intercept	-1156.66	225.04	-5.13982	8.2E-05	-1631.46	-681.871	-1631.46	-681.871
Life Expectancy	14.2147	2.80399	5.06945	9.5E-05	8.29878	20.1306	8.29878	20.1306
% Severe Housing Problems	4.44839	1.51249	2.9411	0.00913	1.25731	7.63947	1.25731	7.63947
% Black	1.10323	0.87738	1.25742	0.2256	-0.74787	2.95433	-0.74787	2.95433

Figure 18: Connecticut Datasets

Connecticut County	Deaths per 100,000 (ACTUALS)	Life Expectancy	% Black	Deaths per 100,000 (MODEL)	Absolute Difference (Model - Actuals)
	57.7	**13.3**	**5.1**		13.3
Fairfield County	106.6	82.7	11.0	107.0	0.4
Hartford County	99.1	80.3	13.2	86.7	12.4
Litchfield County	57.1	80.4	1.6	28.8	28.3
Middlesex County	66.5	81.1	4.8	54.0	12.5
New Haven County	78.3	80.1	12.8	82.5	4.2
New London Count	18.9	79.9	5.7	42.4	23.6
Tolland County	30.5	81.6	3.3	52.5	22.0
Windham County	4.3	78.7	1.9	7.3	3.0

Regression Statistics	
Multiple R	0.8808012
R Square	0.7758108
Adjusted R S	0.6861352
Standard Err	20.870153
Observations	8

ANOVA

	df	*SS*	*MS*	*F*	*Significance F*
Regression	2	7536.3752	3768.1876	8.6512975	0.0237978
Residual	5	2177.8164	435.56329		
Total	7	9714.1916			

	Coefficients	*Standard Erro*	*t Stat*	*P-value*	*Lower 95%*	*Upper 95%*	*Lower 95.0%*	*Upper 95.0%*
Intercept	-1046.3068	546.33982	-1.915121	0.1136374	-2450.7181	358.10436	-2450.718054	358.104364
Life Expectai	13.262672	6.8123085	1.946869	0.1091067	-4.2489243	30.774269	-4.248924336	30.77426867
% Black	5.1425669	1.6857731	3.0505689	0.0284059	0.8091492	9.4759846	0.80914922	9.475984584

Figure 19: Massachusetts Datasets

County	Deaths per 100,000 (ACTUALS)	Average Daily PM2.5	% Black	Deaths per 100,000 (MODEL)	Absolute Difference (Model - Actuals)
	47.0	27.3	2.3		13.0
Barnstable County	22.5	6.7	3.1	19.3	3.2
Berkshire County	28.6	7.7	3.1	46.7	18.1
Bristol County	37.2	6.9	4.6	28.2	9.1
Dukes County	5.8	6.0	4.1	2.4	3.4
Essex County	66.9	7.8	3.4	50.0	16.9
Franklin County	52.4	7.7	1.3	42.5	9.9
Hampden County	88.6	8.2	7.7	70.9	17.7
Hampshire County	29.2	8.0	2.9	54.4	25.2
Middlesex County	63.9	8.5	5.2	73.4	9.5
Nantucket County	-	6.0	9.5	14.7	14.7
Norfolk County	81.8	8.1	7.1	66.6	15.1
Plymouth County	65.8	7.2	10.6	50.2	15.6
Suffolk County	75.8	7.6	20.3	83.3	7.4
Worcester County	39.9	7.9	4.7	55.8	15.9

Regression Statistics	
Multiple R	0.848034
R Square	0.719161
Adjusted R Square	0.6681
Standard Error	16.09356
Observations	14

ANOVA

	df	*SS*	*MS*	*F*	*ignificance F*
Regression	2	7295.692	3647.846	14.0842	0.000926
Residual	11	2849.03	259.0027		
Total	13	10144.72			

	Coefficients	*andard Erro*	*t Stat*	*P-value*	*Lower 95%*	*Upper 95%*	*Lower 95.0%*	*Upper 95.0%*
Intercept	-170.993	43.30448	-3.94863	0.002278	-266.306	-75.6806	-266.3056673	-75.68064548
Average Daily PM2.5	27.34238	5.69734	4.799149	0.000554	14.80262	39.88215	14.80262269	39.88214529
% Black	2.283718	0.921812	2.477422	0.030714	0.254823	4.312614	0.254823134	4.312613726

Figure 20: Louisiana Datasets

County	Deaths per 100,000 (ACTUALS)	Life Expectancy	% Black	% Hispanic	Primary Care Physicians Ratio	Deaths per 100,000 (MODEL)	Absolute Difference (Model - Actuals)
(369.9)	37.4	4.5	1.6	5.3	8.0		22.1
County	Deaths per 100k						
Acadia	17.7	75.4	17.9	2.7	0.23	12.3	5.4
Allen	35.1	74.9	22.3	2.3	0.90	19.8	15.3
Ascension	36.3	78.7	23.2	5.7	0.11	50.4	14.0
Assumption	45.7	77.6	29.4	3.1	1.15	49.6	3.9
Avoyelles	17.4	73.8	29.6	2.0	0.62	22.4	4.9
Beauregard	10.7	74.4	12.0	3.8	0.08	2.9	7.8
Bienville	128.4	74.4	41.5	1.9	1.09	47.4	81.0
Bossier	15.0	77.9	22.5	6.8	-0.01	49.8	34.9
Caddo	53.3	74.9	49.4	2.9	-2.79	36.1	17.2
Calcasieu	18.7	75.1	25.4	3.8	-1.32	15.8	2.8
Caldwell	-	73.5	15.8	3.2	0.49	4.9	4.9
Cameron	-	82.0	4.0	4.3	0.54	30.2	30.2
Catahoula	21.1	73.6	30.5	1.7	1.27	27.0	5.9
Claiborne	57.4	75.9	51.6	1.5	0.20	60.4	3.0
Concordia	20.8	73.0	39.6	1.5	0.49	30.8	10.0
De Soto	47.3	76.1	35.7	3.0	0.95	50.2	2.9
East Baton Roug	38.9	76.1	46.5	4.2	-1.96	50.5	11.7
East Carroll	-	72.7	68.0	2.5	0.03	75.4	75.4
East Feliciana	99.3	73.5	42.5	1.7	0.46	38.3	61.0
Evangeline	3.0	73.7	27.4	3.8	-0.76	17.3	14.3
Franklin	20.0	73.9	31.5	1.7	0.32	22.0	2.0
Grant	-	75.5	15.2	5.2	1.32	30.1	30.1
Iberia	37.2	74.7	32.1	4.3	-0.17	36.4	0.8
Iberville	107.7	75.5	48.3	2.7	0.37	61.1	46.5
Jackson	25.4	76.3	28.4	1.8	0.93	33.2	7.8
Jefferson	90.6	77.3	26.8	14.9	-1.87	82.0	8.6

County	Deaths per 100,000 (ACTUALS)	Life Expectancy	% Black	% Hispanic	Primary Care Physicians Ratio	Deaths per 100,000 (MODEL)	Absolute Difference (Model - Actuals)
Jefferson Davis	19.1	73.9	16.4	2.5	-0.07	(0.5)	19.6
Lafayette	8.6	78.5	26.4	4.6	-2.46	28.1	19.5
Lafourche	58.4	77.3	13.4	4.4	-0.15	19.1	39.3
La Salle	-	75.4	12.0	2.9	-0.68	(3.3)	3.3
Lincoln	23.5	77.8	40.3	3.2	-0.86	51.9	28.4
Livingston	14.9	76.5	6.5	3.8	0.93	10.7	4.2
Madison	-	72.4	62.4	2.2	0.29	66.1	66.1
Morehouse	20.1	71.9	47.7	1.4	0.30	36.8	16.7
Natchitoches	21.0	74.7	41.0	2.4	-0.53	37.8	16.8
Orleans	118.7	76.9	59.1	5.6	-2.03	80.5	38.1
Ouachita	16.3	74.9	37.3	2.2	-1.58	23.4	7.0
Plaquemines	73.3	78.2	20.7	7.8	1.00	62.1	11.2
Pointe Coupee	78.2	75.7	35.3	2.9	0.44	43.4	34.8
Rapides	8.5	74.4	31.7	3.2	-1.50	18.0	9.5
Red River	71.1	72.8	39.4	2.4	0.75	36.7	34.4
Richland	5.0	74.8	35.4	2.3	-1.01	24.6	19.7
Sabine	4.2	76.4	16.7	4.0	1.18	28.7	24.5
St. Bernard	42.3	74.9	22.9	10.2	0.82	61.9	19.5
St. Charles	81.0	77.8	26.0	6.3	0.34	55.3	25.7
St. Helena	9.9	74.1	51.9	1.9	0.91	60.5	50.6
St. James	94.8	76.1	48.8	1.7	0.75	62.3	32.5
St. John the Bapt	177.4	74.6	57.0	6.3	0.59	91.6	85.8
St. Landry	59.7	72.9	41.3	2.3	-1.04	25.1	34.6
St. Martin	37.4	75.8	29.7	3.0	1.02	40.2	2.7
St. Mary	50.7	75.4	31.4	7.1	-0.32	52.3	1.6
St. Tammany	48.0	78.6	12.3	5.7	-1.49	19.5	28.5
Tangipahoa	20.0	74.6	30.1	4.4	0.31	37.1	17.1
Tensas	-	75.1	53.9	2.2	0.00	62.6	62.6
Terrebonne	32.6	75.6	19.0	5.1	0.07	25.7	6.9
Union	36.2	75.5	24.8	4.6	1.32	41.8	5.6
Vermilion	3.4	76.8	14.2	3.7	0.43	18.9	15.6
Vernon	4.2	74.9	13.8	9.4	0.44	40.0	35.8

Figure 21: Louisiana Datasets (continued)

County	Deaths per 100,000 (ACTUALS)	Life Expectancy	% Black	% Hispanic	Primary Care Physicians Ratio	Deaths per 100,000 (MODEL)	Absolute Difference (Model - Actuals)
Washington	52.0	72.5	30.1	2.4	-0.17	13.4	38.6
Webster	10.4	73.6	34.0	2.0	-0.71	17.9	7.5
West Baton Roug	90.7	78.1	39.4	3.2	1.07	66.9	23.7
West Carroll	-	74.3	15.6	4.0	0.60	12.8	12.8
West Feliciana	38.5	77.9	44.3	1.6	-1.12	47.6	9.0
Winn	14.4	74.1	30.6	2.2	-0.51	17.4	3.0

Regression Statistics	
Multiple R	0.574296
R Square	0.329816
Adjusted R Square	0.28438
Standard Error	31.38031
Observations	64

ANOVA

	df	*SS*	*MS*	*F*	*ignificance F*
Regression	4	28591.97	7147.993	7.258881	8E-05
Residual	59	58098.71	984.7239		
Total	63	86690.68			

	Coefficients	*tandard Err*	*t Stat*	*P-value*	*Lower 95%*	*Upper 95%*	*Lower 95.0%*	*Upper 95.0%*
Intercept	-369.92	180.2507	-2.05226	0.04459	-730.601	-9.239670291	-730.6011442	-9.239670291
Life Expectancy	4.47911	2.371213	1.888953	0.063816	-0.26568	9.223896217	-0.265676428	9.223896217
% Black	1.56771	0.313451	5.001452	5.42E-06	0.940496	2.194923471	0.940495878	2.194923471
% Hispanic	5.274813	1.886269	2.796427	0.006964	1.500398	9.049228929	1.500397886	9.049228929
Primary Care Physicians Ratio	7.95318	4.167582	1.908344	0.061216	-0.38613	16.29249232	-0.386132214	16.29249232

Figure 21: Michigan Datasets

County	Deaths per 100,000 (ACTUALS)	Median Household Income	% Black	Deaths per 100,000 (MODEL)	Absolute Difference (Model - Actuals)
(10.4)	11.4	0.0	2.0		9.7
Alcona	0	39862	0.5	1.2	1.2
Alger	0	47441	7.2	16.9	16.9
Allegan	1.7	62247	1.3	8.9	7.2
Alpena	28.2	42792	0.5	2.0	26.1
Antrim	-	56456	0.3	5.2	5.2
Arenac	6.7	42540	0.5	1.9	4.8
Baraga	-	43435	7.9	17.2	17.2
Barry	1.6	64121	0.6	7.9	6.3
Bay	8.7	50576	1.7	6.5	2.3
Benzie	-	51905	0.6	4.7	4.7
Berrien	14.3	51509	14.6	32.9	18.5
Branch	4.6	50477	2.2	7.4	2.8
Calhoun	12.7	48413	10.8	24.3	11.7
Cass	3.9	55978	5.0	14.7	10.8
Charlevo	3.8	54491	0.5	5.1	1.3
Cheboyga	4.0	48589	0.6	3.8	0.1
Chippew	-	47380	6.6	15.6	15.6
Clare	6.5	39394	0.7	1.5	4.9
Clinton	12.6	70967	1.9	12.4	0.2
Crawford	28.5	46930	0.8	3.8	24.7
Delta	5.6	49292	0.4	3.5	2.1
Dickinso	7.9	52348	0.5	4.6	3.4
Eaton	5.4	66716	6.7	20.9	15.5
Emmet	6.0	58891	0.7	6.8	0.8
Genesee	52.7	48370	20.0	43.0	9.7
Gladwin	3.9	44635	0.5	2.5	1.4
Gogebic	7.2	39934	3.9	8.1	1.0
Grand Tr	5.4	64591	0.7	8.2	2.9
Gratiot	4.9	48988	5.8	14.4	9.5
Hillsdale	46.0	50388	0.6	4.3	41.7
Houghto	-	42282	0.9	2.7	2.7
Huron	3.2	45817	0.6	2.9	0.3
Ingham	5.5	52837	11.5	27.0	21.5
Ionia	3.1	61667	4.5	15.1	12.0
Iosco	31.8	41256	0.7	2.0	29.8
Iron	-	40959	0.4	1.4	1.4
Isabella	10.0	47056	2.6	7.4	2.6
Jackson	15.8	52047	7.9	19.5	3.8
Kalamaz	9.4	55881	11.4	27.6	18.2
Kalkaska	11.1	46068	0.6	3.1	8.0

County	Deaths per 100,000 (ACTUALS)	Median Household Income	% Black	Deaths per 100,000 (MODEL)	Absolute Difference (Model - Actuals)
Kent	6.2	61675	9.7	25.6	19.4
Keweena	-	51641	0.3	4.0	4.0
Lake	-	36044	8.4	16.2	16.2
Lapeer	33.1	65209	1.1	9.3	23.8
Leelanau	-	67349	0.6	8.7	8.7
Lenawee	2.0	55762	2.6	9.7	7.6
Livingsto	10.4	84048	0.6	13.2	2.8
Luce	-	43196	11.5	24.4	24.4
Mackina	-	46645	3.1	8.3	8.3
Macomb	78.0	62191	12.1	30.6	47.4
Manistee	-	47401	3.4	9.2	9.2
Marquett	13.5	54093	1.5	7.1	6.4
Mason	-	51083	0.8	4.8	4.8
Mecosta	4.6	45804	2.7	7.4	2.8
Menomi	-	46486	0.6	3.3	3.3
Midland	9.6	58057	1.3	7.7	1.9
Missauk	6.6	48413	0.5	3.5	3.1
Monroe	10.6	62522	2.5	11.3	0.7
Montcalm	1.6	50396	2.4	8.0	6.4
Montmor	-	40650	0.5	1.4	1.4
Muskego	10.9	49960	13.8	31.0	20.0
Newaygo	-	48860	1.0	4.7	4.7
Oakland	64.0	80319	13.8	38.9	25.1
Oceana	3.8	50374	1.0	5.1	1.3
Ogemaw	-	41201	0.3	1.2	1.2
Ontonag	-	40798	0.3	1.1	1.1
Osceola	-	45607	0.8	3.4	3.4
Oscoda	-	38927	0.5	0.9	0.9
Otsego	36.5	51292	0.5	4.3	32.2
Ottawa	5.8	70420	1.6	11.5	5.7
Presque	-	45502	0.6	3.0	3.0
Roscomm	-	38613	0.5	1.0	1.0
Saginaw	41.5	48805	18.5	40.1	1.3
St. Clair	14.5	56557	2.4	9.6	4.9
St. Josep	1.6	52689	2.4	8.5	6.9
Sanilac	12.1	46184	0.5	3.0	9.1
Schoolcra	-	44887	0.4	2.5	2.5
Shiawass	23.5	54149	0.6	5.3	18.2
Tuscola	32.5	51113	1.2	5.7	26.8
Van Bure	2.6	57363	3.7	12.4	9.7
Washten	21.8	72267	12.0	33.2	11.4
Wayne	115.9	46440	38.3	79.6	36.3
Wexford	5.9	46396	0.6	3.2	2.8

Regression Statistics	
Multiple R	0.665215
R Square	0.442511
Adjusted R Square	0.428574
Standard Error	14.33978
Observations	83

ANOVA

	df	*SS*	*MS*	*F*	*Significance F*
Regression	2	13057.59	6528.797	31.75031	7.0705E-11
Residual	80	16450.35	205.6294		
Total	82	29507.94			

	Coefficients	*tandard Err*	*t Stat*	*P-value*	*Lower 95%*	*Upper 95%*	*Lower 95.0%*	*Upper 95.0%*
Intercept	-10.3631	8.786605	-1.17942	0.241725	-27.8490409	7.12276138	-27.8490409	7.12276138
Median Household Income	0.000266	0.000168	1.583137	0.117336	-6.8348E-05	0.00060016	-6.8348E-05	0.00060016
% Black	2.027041	0.26483	7.654109	3.84E-11	1.50001184	2.55407066	1.50001184	2.55407066

Figure 22: Pennsylvania Datasets

County	Deaths per 100,000 (ACTUAL)	% Severe Housing Problems	% Hispanic	Deaths per 100,000 (MODEL)	Absolute Difference (Model - Actuals)
Adams	0.5	12	7.1	1.1	0.6
Allegheny	0.9	14	2.2	1.6	0.7
Armstrong	0.5	10	0.8	0.4	0.0
Beaver	4.4	11	1.6	0.6	3.9
Bedford	0.2	12	1.2	0.9	0.7
Berks	3.5	15	21.9	2.1	1.4
Bradford	0.3	11	1.5	0.7	0.4
Bucks	4.5	15	5.5	2.0	2.6
Butler	0.3	11	1.6	0.6	0.3
Cambria	0.1	12	1.7	1.1	1.1
Carbon	2.3	14	5.1	1.7	0.7
Centre	0.1	18	3.0	2.9	2.8
Chester	2.9	14	7.6	1.8	1.1
Clarion	0.3	13	1.0	1.3	1.0
Columbia	2.4	12	2.9	1.2	1.3
Cumberland	1.0	12	4.1	0.9	0.1
Dauphin	1.0	14	9.6	1.7	0.6
Delaware	5.6	17	3.9	2.6	3.0
Erie	0.1	14	4.4	1.7	1.6
Fayette	0.3	13	1.2	1.3	0.9
Franklin	0.6	11	5.9	0.8	0.2
Greene	0.3	10	1.6	0.5	0.3
Indiana	0.6	16	1.3	2.4	1.8
Juniata	0.4	11	4.0	0.6	0.2
Lackawanna	4.9	16	8.1	2.4	2.5
Lancaster	2.6	15	10.8	2.1	0.6
Lawrence	0.8	13	1.5	1.5	0.6
Lebanon	1.1	13	13.8	1.4	0.3
Lehigh	2.6	17	25.4	2.5	0.1
Luzerne	3.3	14	12.9	1.8	1.5
Lycoming	0.4	14	2.1	1.6	1.2

County	Deaths per 100,000 (ACTUAL)	% Severe Housing Problems	% Hispanic	Deaths per 100,000 (MODEL)	Absolute Difference (Model - Actuals)
Mercer	0.1	12	1.6	1.1	1.0
Monroe	3.5	19	16.6	3.4	0.2
Montgomery	5.3	15	5.3	1.9	3.5
Northampton	3.8	15	13.8	1.9	1.9
Perry	0.2	10	2.1	0.3	0.1
Philadelphia	4.0	23	15.2	4.6	0.7
Pike	3.0	18	11.4	2.9	0.2
Schuylkill	0.5	12	4.8	0.9	0.4
Snyder	0.2	13	2.4	1.2	1.0
Somerset	0.1	11	1.6	0.7	0.5
Susquehanna	2.7	13	1.7	1.3	1.4
Tioga	0.2	13	1.3	1.2	1.0
Union	0.2	12	6.0	1.1	0.9
Washington	0.1	10	1.8	0.5	0.4
Wayne	1.0	15	4.7	2.0	1.1
Westmoreland	0.8	10	1.2	0.4	0.4
Wyoming	0.7	13	1.9	1.4	0.7
York	0.2	13	7.9	1.3	1.1

Regression Statistics	
Multiple R	0.696038114
R Square	0.484469056
Adjusted R Square	0.462054667
Standard Error	1.218155526
Observations	49

ANOVA

	df	*SS*	*MS*	*F*	*Significance F*
Regression	2	64.14674386	32.07337193	21.61419877	2.40912E-07
Residual	46	68.25953277	1.483902886		
Total	48	132.4062766			

	Coefficients	*Standard Error*	*t Stat*	*P-value*	*Lower 95%*	*Upper 95%*	*Lower 95.0%*	*Upper 95.0%*
Intercept	-6.275983673	1.209279766	-5.189852547	4.63615E-06	-8.710137592	-3.841829755	-8.710137592	-3.841829755
% Severe Housing Problems	0.316458147	0.066257067	4.776217276	1.85511E-05	0.183089589	0.449826705	0.183089589	0.449826705
Median Household Income	6.04418E-05	1.57929E-05	3.827162119	0.000389568	2.86524E-05	9.22312E-05	2.86524E-05	9.22312E-05
