# Supplementary material for: Predicting Health Disparities in Regions at Risk of Severe Illness to Inform Health Care Resource Allocation During Pandemics: Observational Study
Source: JMIRx Med. 2020 Dec 2;1(1):e22470. doi: 10.2196/22470 (PMC7924701; doi:10.2196/22470)
Supplement: Multimedia Appendix 2 [file med_v1i1e22470_app2.docx]

**Other datasets referenced in this research:**

- Tableau Dashboard on Vulnerability:   <https://c19hcc.org/resource/vulnerable-population>
- World Health Organization (WHO) dashboard: <https://who.sprinklr.com/>
- Full list of datasets: <https://cgdv.github.io/challenges/COVID-19/datasource/>
- Uninsured data: <https://www.census.gov/data-tools/demo/sahie/#/data_2013>
- COVID-19 deaths by ethnicity: <https://www.apmresearchlab.org/covid/deaths-by-race>
- FIPS to Zip code mapping: <https://data.world/niccolley/us-zipcode-to-county-state/workspace/file?filename=ZIP-COUNTY-FIPS_2018-03.csv>
- Nursing Home (Medicare) by Zip code: <https://data.medicare.gov/data/nursing-home-compare>
- Johns Hopkins tracking of states that currently provide COVID data by ethnicity ( <https://coronavirus.jhu.edu/data/racial-data-transparency> )

**Other research papers evaluated during the course of this research:**

- Research paper based on China data (complicated paper, but some good ideas): <https://doi.org/10.1101/2020.03.13.20035238doi>
- Research paper on socio-economic status and cardio-vascular disease (not directly applicable to COVID-19, but interesting): <https://www.ncbi.nlm.nih.gov/pmc/articles/PMC1694190/>
- FastCompany – excellent article with many ideas for hypotheses to test: <https://www.fastcompany.com/90479231/9-maps-that-show-which-areas-could-be-more-vulnerable-to-the-covid-19-pandemic>
- Fivethirtyeight article: <https://fivethirtyeight.com/features/the-young-americans-most-vulnerable-to-covid-19-are-people-of-color-and-the-working-class/>
- The Conversation article: <https://theconversation.com/covid-19-is-hitting-black-and-poor-communities-the-hardest-underscoring-fault-lines-in-access-and-care-for-those-on-margins-135615>
- HealthAffairs article: <https://www.healthaffairs.org/do/10.1377/hblog20200319.757883/full/>
- Vox article: <https://www.vox.com/identities/2020/4/7/21211849/coronavirus-black-americans>
- FastCompany article on interesting data tool for Urban Footprint: <https://www.fastcompany.com/90481120/this-tool-is-helping-cities-find-the-neighborhoods-most-vulnerable-to-coronavirus>
- Persistent geographic variations in availability and quality of nursing home care in the United States: 1996 to 2016 | BMC Geriatrics: <https://bmcgeriatr.biomedcentral.com/articles/10.1186/s12877-019-1117-z>
- Do Lockdowns Save Many Lives? In Most Places, the Data Say No: <https://www.wsj.com/articles/do-lockdowns-save-many-lives-is-most-places-the-data-say-no-11587930911?emailToken=f098d16b875951b5c8f988a12e639f1eOHpwB2N8i9ta8xko7k29ejymFwfBHRJxh+pWaTu1FkXQvn85QSXeuxBX0JI/nDe1DCUBUTnLl2Lah6/ggNGXoFlA205HsvxNzx0X69Dkjf50N1DlSdrruzjxIT/8BzYe&reflink=article_email_share>
- NYC’s Deaths Mirror Patterns Elsewhere: <https://www.wsj.com/articles/new-york-citys-coronavirus-deaths-match-demographics-in-other-hot-spots-11587214800?emailToken=431a69ce96c62d7963b263078ba230737i1M5JE3rrp14zRmD2CWM3JsOVaJyWmS5o0CyI69f0ZsVl8dolZ1yLPaoZtPrXy1OTsEJJYygy4rPaqPY3w2uK45f2aAFtPA007//tC6A3ZlzBkmToXjwuLlllX2LEAjTb2Pf9cH54YlNUPzTwSG2Q%3D%3D&reflink=article_email_share>
- New COVID-19 Community Vulnerability Map Uses Social Determinants of Health to Identify Populations At Greater Risk: <https://hitconsultant.net/2020/03/24/covid-19-community-vulnerability-map/#.XqS3EzNKhPY>
- What Does and Does Not Correlate with COVID-19 Death Rates: <https://www.nber.org/papers/w27391>
